# Precentral Gyrus Connectivity in Auditory Verbal Hallucinations: A Resting-State Functional MRI Study

**DOI:** 10.31083/AP45829

**Published:** 2025-12-18

**Authors:** Vyara Zaykova, Ferihan Popova, Sevdalina Kandilarova, Drozdstoy Stoyanov

**Affiliations:** ^1^Department of Anatomy, Histology and Embryology, Faculty of Medicine, Medical University of Plovdiv, 4000 Plovdiv, Bulgaria; ^2^Department of Psychiatry and Medical Psychology, Research Institute and SRIPD-MUP, Translational and Computation Neuroscience Group, Faculty of Medicine, Medical University of Plovdiv, 4000 Plovdiv, Bulgaria

**Keywords:** schizophrenia, auditory verbal hallucinations, functional magnetic resonance imaging, precentral gyrus

## Abstract

**Background:**

The objective of this study was to analyze the functional connectivity (FC) of the precentral gyrus (PCG) bilaterally in a sample of patients with schizophrenia experiencing chronic auditory verbal hallucinations (AVH) including a control cohort of healthy volunteers.

**Methods:**

A total of 105 subjects underwent resting-state functional magnetic resonance imaging (MRI) scanning, including 63 healthy control individuals (HC) and 42 schizophrenia patients experiencing AVH. A comparative approach was used to analyze the FC of the PCG bilaterally.

**Results:**

The present study detected increased resting-state FC (rsFC) involving the right PCG and three clusters distributed bilaterally across the frontal cortex, the supplementary motor area (SMA), paracingulate gyrus and the anterior cingulate gyrus (ACC), as well as hypoconnectivity between the right PCG and the lingual gyrus – bilaterally and the left occipital fusiform gyrus in schizophrenia as compared to HC. Furthermore, we observed hyperconnectivity between the left PCG and four clusters, including right paracingulate gyrus, ACC, right frontal pole (FP), precuneus, right pre- and postcentral gyri, right superior frontal gyrus (SFG), and right SMA. In addition, the patient group demonstrated hypoconnectivity between the left PCG and the right occipital pole, right lingual gyrus, right lateral occipital cortex, as well as the right cerebellar crus 1.

**Conclusions:**

In the present study we observed a lateralized impairment in rsFC between the explored seeds and specific cortical and subcortical regions in schizophrenia. These alterations might contribute to the neurobiological pathways involved in schizophrenia pathogenesis with a focus on higher hallucination proneness.

## Main Points

1. Widespread hyperconnectivity between the precentral gyrus and frontal-cingulate regions in schizophrenia:

Both precentral gyri showed heightened resting state functional connectivity (rsFC) with frontal areas (e.g., superior/middle frontal gyri, frontal pole, anterior cingulate cortex (ACC), indicating altered motor-frontal integration in schizophrenia.

2. Reduced connectivity with visual and occipital regions in patients with schizophrenia:

Patients showed reduced rsFC between the precentral gyrus and visual regions (e.g., lingual gyrus, occipital cortex, cerebellum), suggesting impaired sensory-motor integration.

3. Functional connectivity alterations reflect dysregulation of motor, cognitive control, and sensory processing networks:

The pattern of hyperconnectivity with executive and motor-related regions, alongside hypoconnectivity with visual processing areas, supports the hypothesis of widespread network dysconnectivity in schizophrenia, potentially underlying key clinical symptoms such as motor disturbances and perceptual abnormalities.

## 1. Introduction 

Schizophrenia is a debilitating psychotic disorder that is characterized by a variety of symptoms, with auditory verbal hallucinations (AVH) being the most commonly observed [1, 2]. The term AVH refers to a phenomenon of hearing voices in the absence of external auditory stimuli [3]. The mechanisms involved in the occurrence of AVH are not entirely elucidated. It has been suggested that the development of AVH is a consequence of altered cerebral processes that are incapable of distinguishing between internal mental activity and that triggered by external sensory input [4].

The development of functional magnetic resonance imaging (fMRI) has enabled non-invasive investigation of cognitive processes. This contemporary imaging technique is capable of detecting fluctuations in the blood oxygen level-dependent (BOLD) signal, which are indicative of an elevated blood supply to the neurons upon their activation. The variations in the BOLD response form the basis of fMRI, commonly utilized to generate visualizations illustrating distinct cerebral regions that are engaged during the performance of particular tasks or in response to external stimulations. In contrast, resting-state fMRI (rs-fMRI) is obtained without external stimuli or certain tasks, when the subject is at rest. The principal purpose of rs-fMRI is to observe natural fluctuations in the BOLD response. One approach to analyze the rs-fMRI data is functional integration. This method is used to identify functional connectivity (FC) between different parts of the brain [5]. FC examines the functional relationship between discrete brain regions by statistical dependence of the time series of activity [6].

The findings of prior fMRI studies have reported that abnormalities implicated in AVH are not completely associated with particular brain regions or functional networks, but rather, the focus should be on the FC of a series of regions distributed across multiple functional systems [7, 8, 9]. Disturbed FC has been reported between frontal and temporo-parietal regions [10], as well as between nodes of the default mode network (DMN), salience network (SN) and central executive network (CEN) in AVH [11]. Previous studies have also demonstrated anomalies in the FC of the precentral gyrus (PCG) in patients with schizophrenia [12, 13]. This region is part of a neural system that plays a crucial role in the motor expression of language and auditory monitoring and feedback of speech [11]

Moreover, in a study by Shinn *et al*. [13], the aberrant FC between the primary auditory cortex and the PCG exhibited a direct correlation with the degree of AVH symptoms. Furthermore, positive correlation was established the degree of coactivation within the motor network and the presence of AVH in patients with schizophrenia [8]. Conversely, according to a study by Chang *et al*. [12], abnormal bilateral connectivity of the PCG was specific to the non-AVH patients with schizophrenia. However, the findings from prior studies were inconsistent regarding the role of the primary motor cortex in the pathophysiology of AVH. Thus, the aim of our study was to explore the FC of the PCG bilaterally among patients clinically diagnosed with schizophrenia, experiencing chronic AVH in comparison to a control group composed of healthy subjects. According to previous findings, we expected to observe alterations in the FC of the PCG specific to the schizophrenia group.

## 2. Materials and Methods

### 2.1 Subjects

Data from resting-state fMRI were acquired from 110 individuals ranging in age from 18 to 61. Following a review of data quality, five cases were excluded from the neuroimaging analysis due to missing demographic or clinical data. The final sample is comprised of 105 participants divided into two groups: HC (n = 63; m/f = 31/32; mean age 36.0 Standard Deviation (SD) ± 12.5) and AVH (n = 42; m/f = 26/16; mean age 35.3 SD ± 12.4).

Participants took part voluntarily without any financial reward. Prior to enrolment in the study, informed written consent was obtained from each subject. The research protocol received approval from the Medical University of Plovdiv Ethical Committee (Ref. No. 1/11.01.2024). All procedures followed the ethical guidelines of the Helsinki Declaration (1964) and its later revisions.

Each participant has been assessed by a physician using the Positive and Negative Symptom Scale (PANSS). All patients met diagnostic criteria for schizophrenia according to Diagnostic and Statistical Manual of Mental Disorders, fourth edition, text revision (DSM-IV TR). The patient sample inclusion depended on the existence of severe auditory verbal hallucinations (P3>3). The non-clinical participants were matched according to sex and age. Neurological diseases or psychiatric conditions, as well as history of brain injury were exclusion criteria for the participants. Metal implants or any other factors contraindicating MRI were additional exclusion criteria applied to both patients and healthy controls (HC).

### 2.2 Procedure for MRI Data Acquisition

The magnetic resonance scanning procedure was performed on a 3T magnetic resonance imaging system (GE Discovery 750w, GE Healthcare Technologies, Chicago, IL, USA). The protocol included high resolution structural scan (Sag 3D T1 FSPGR) with the following parameters: slice thickness 1 mm, matrix size 256 × 256, time of relaxation 7.2 milliseconds, time of echo 2.3 milliseconds .The flip angle was set to 12 degrees for the structural scan. Resting-state functional imaging was performed using 2D Echo Planar Imaging (EPI) with a slice thickness of 3 mm, a matrix size of 64 × 64, a repetition time (TR) of 2 seconds, and an echo time (TE) of 30 milliseconds. A total of 36 slices were acquired at a 90-degree angle, resulting in 160 volumes collected. Participants were instructed to remain relaxed, refrain from engaging in defined thoughts and from opening their eyes throughout the functional imaging session.

### 2.3 Image Processing

The functional data were analyzed with the CONN toolbox (RRID:SCR_009550; https://www.nitrc.org/projects/conn), version 21.a, implemented in Statistical Parametric Maping software (SPM) version 12 (RRID:SCR_007037, https://www.fil.ion.ucl.ac.uk/spm/) and executed in MATLAB R2024a (The MathWorks, Inc. https://www.mathworks.com) on a Windows platform. Image preprocessing followed the toolbox’s default pipeline.

Preprocessing: The preprocessing stages included the following steps: realignment with correction of susceptibility distortion interactions, slice timing correction, outlier detection, direct segmentation and Montreal Neuroimaging Institute (MNI) space normalization, and smoothing. Functional data were realigned using SPM realign & unwarp procedure. Temporal misalignment between different slices of the functional data was corrected following SPM slice-timing correction (STC) procedure. Both functional and anatomical datasets were normalized to the standard MNI template, segmented into gray matter, white matter, and cerebrospinal fluid compartments, and resampled to 2 mm isotropic resolution. This process employed SPM’s unified segmentation and normalization approach, using the default IXI-549 tissue probability maps. Subsequently, functional images were spatially smoothed with an 8 mm full-width at half-maximum (FWHM) Gaussian kernel.

Denoising: Functional data underwent the toolbox’s standard denoising procedure, which involved regressing out potential confounding signals, including white matter and cerebrospinal fluid time series, head-motion parameters with their first derivatives, outlier volumes, session effects and their derivatives, as well as linear trends for each run. Finally, the BOLD time series were temporally filtered using a band-pass range of 0.008–0.09 Hz.

First-level analysis: Seed-based connectivity maps and ROI-to-ROI (region of interest) connectivity matrices (RRC) were estimated characterizing the patterns of functional connectivity with 164 HPC-ICA networks and Harvard-Oxford atlas ROIs. Functional connectivity strength was represented by Fisher-transformed bivariate correlation coefficients from a weighted general linear model (GLM), defined separately for each pair of seed and target areas, modeling the association between their BOLD signal timeseries.

Group-level analyses were conducted using a GLM. At each voxel, an individual GLM was computed, with the voxel-wise first-level connectivity values as the dependent variable and group membership or other subject-specific factors as independent variables. Voxel-level hypotheses were evaluated using multivariate parametric statistics with random-effects across subjects and sample covariance estimation across multiple measurements. Inferences were performed at the level of individual clusters (groups of contiguous voxels). Cluster-level inferences were derived using parametric statistics based on Gaussian Random Field theory. Significance was determined using a voxel-level cluster-forming threshold of *p* < 0.001, combined with a cluster-size threshold corrected for multiple comparisons using the family-wise error rate at *p* < 0.05.

### 2.4 Statistical Analysis 

The socio-demographic variables were analyzed with IBM SPSS Statistics, Version 28.0 (IBM Corp., Armonk, NY, USA). Differences between groups were assessed using the independent samples *t*-test for continuous variables and the chi-square test for categorical variables. A *p*-value below 0.05 was considered statistically significant.

## 3. Results

### 3.1 Social Characteristics and Clinical Data

There was no statistically significant difference concerning age or sex between the patients with AVH and the healthy controls. The two groups differed significantly in terms of educational background, which was expected considering the early clinical manifestation of the condition. Consistent with expectations, PANSS scores were markedly elevated in the patient group compared to controls (Table 1).

**Table 1.** **Demographic and clinical characteristics**.

	P	HC	Statistical significance
	(N = 42)	(N = 63)	
Age	35.3 ± 12.4	36.0 ± 12.5	0.770^a^
(mean, SD)			
Sex (M/F)	26/16	31/32	0.234^b^
^1^Education	8/26/6	1/33/29	*0.0001^b^
(primary/secondary/higher)			
^2^PANSSP3 score	5.1 ± 0.6	1.0 ± 0.0	*0.0001^a^
(mean, SD)			
^3^PANSSP score	21.0 ± 3.6	7.1 ± 0.3	*0.0001^a^
(mean, SD)			
^4^PANSSG score	35.3 ± 7.4	16.8 ± 1.0	*0.0001^a^
(mean, SD)			
^5^PANSSN score	16.9 ± 4.3	6.0 ± 0.0	*0.0001^a^
(mean, SD)			
PANSS score Total	73.1 ± 10.8	29.8 ± 1.1	*0.0001^a^
(mean, SD)			
Age at onset (mean, SD)	25.9 ± 9.6	-	
Illness duration in months (mean, SD)	117.8 ± 102.1	-	

P, patients; HC, healthy controls; SD, standard deviation; M, male; F, female. ^a^ Student’s *t*-test, ^b^ χ^2^ - test, * *p* < 0.05, ^1^Two of the patients were excluded from the analysis due to missing data on education; ^2^PANSSP3 score – AVH – Auditory Verbal Hallucinations; ^3^PANSSP score – Positive Symptoms subscale score; ^4^PANSSG score – General Psychopathology subscale score; ^5^PANSSN score – Negative Symptoms subscale score.

### 3.2 Right Precentral Gyrus Seed-Based Functional Connectivity

Group comparisons indicated that patients with schizophrenia exhibited significantly enhanced rsFC between the right PCG and three clusters relative to healthy participants. The first one was comprised of the right superior frontal gyrus (rSFG), supplementary motor cortex (SMA) - bilaterally and the right middle frontal gyrus (rMFG). The second cluster involved the right frontal pole, the right paracingulate (PC) gyrus and the anterior cingulate cortex (ACC). The third cluster showed increased connection between the right PCG and the rSFG.

**Fig. 1.**
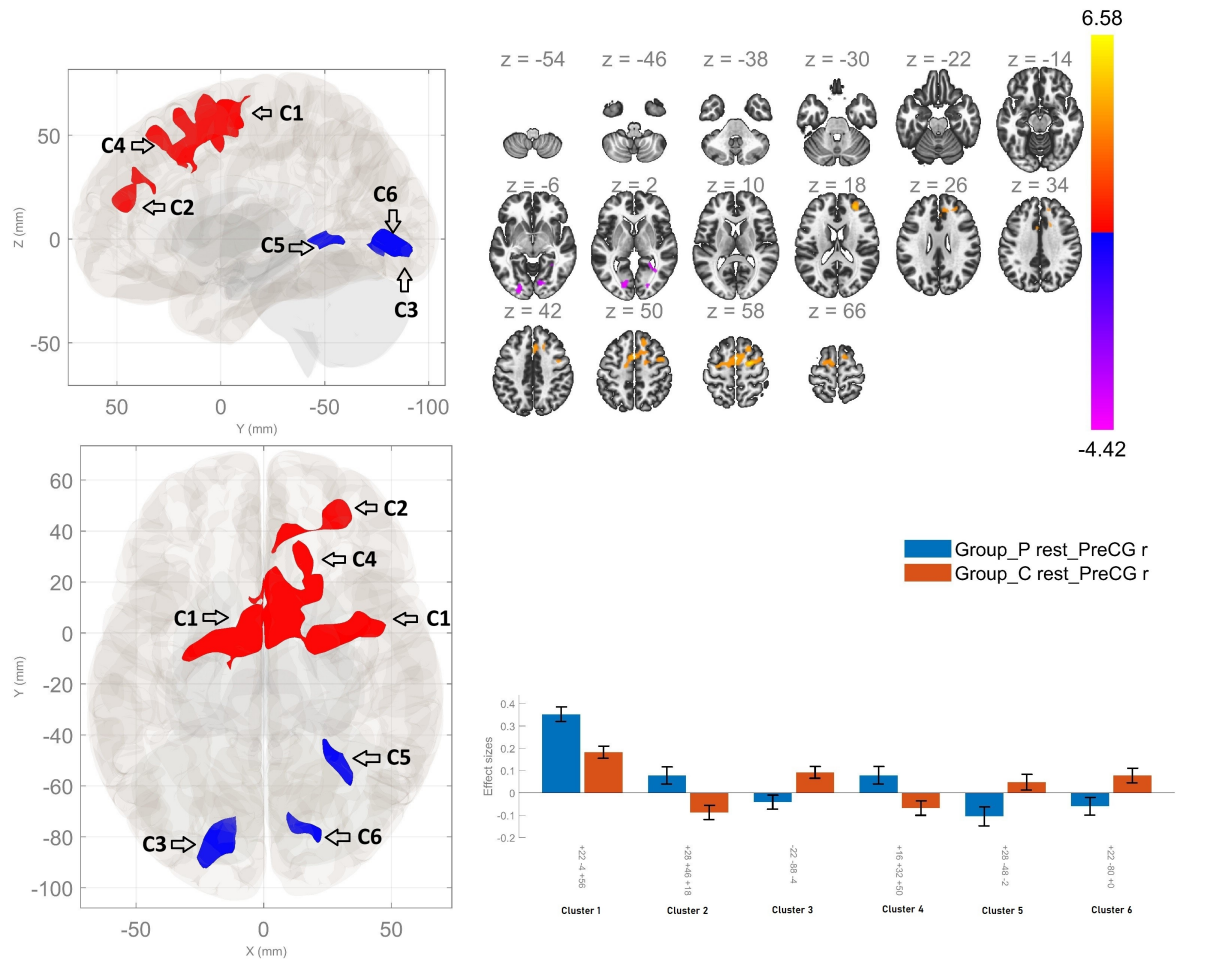
**Resting-state functional connectivity differences of the right precentral gyrus seed between patients with schizophrenia and healthy controls (*p* < 0.05, cluster-level Family-Wise Error (FWE) corrected)**. Patients showed increased resting-state functional connectivity (red areas) with the frontal cortex (cluster 1: +22 –4 +56), right frontal pole and cingulate cortices (cluster 2: +28 +46 +18), and right superior frontal gyrus (cluster 4: +16 +32 +50), and decreased resting-state functional connectivity (blue areas) with the left lingual and occipital fusiform gyri (cluster 3: –22 –88 –4), right lingual gyrus (cluster 5: +28 –48 –2), and right lingual/occipital fusiform gyri (cluster 6: +22 –80 +0).

Conversely, the analyses showed hypoconnectivity between the right PCG and the bilateral lingual gyri, as well as between the analyzed region and the bilateral occipital fusiform gyri within the patient group in contrast to healthy controls (Fig. 1, Table 2). Additionally, we repeated the analyses including the level of education as a covariate of no interest. The results did not change substantially in terms of number of significant clusters, the regions in them and the peak coordinates. There was a non-significant change in the size of the clusters — a reduction amounting to a couple of voxels.

**Table 2.** **Functional connectivity differences of the Right Precentral Gyrus seed between patients with schizophrenia and healthy controls**.

Between-group contrast	MNI coordinates x, y, z	Cluster-size	Cluster-threshold (*p* < 0.05, FWE)	Regions within the cluster
P>HC	+22 –04 +56	1943	0.0001	Bilateral Superior Frontal Gyrus; Bilateral Supplementary Motor Area; Right Middle Frontal Gyrus; Right Paracingulate Gyrus
	+28 +46 +18	350	0.0001	Right Frontal pole; Right Paracingulate Gyrus; Anterior Cingulate Gyrus
	+16 +32 +50	189	0.002	Right Superior Frontal Gyrus
HC>P	–22 –88 –04	296	0.0001	Left Lingual Gyrus; Left Occipital Fusiform Gyrus
	+28 –48 –02	151	0.008	Right Lingual Gyrus
	+22 –80 +00	115	0.032	Right Lingual Gyrus; Right Occipital Fusiform Gyrus

MNI, Montreal Neurological Institute.

### 3.3 Left Precentral Gyrus Seed-Based Functional Connectivity

**Fig. 2.**
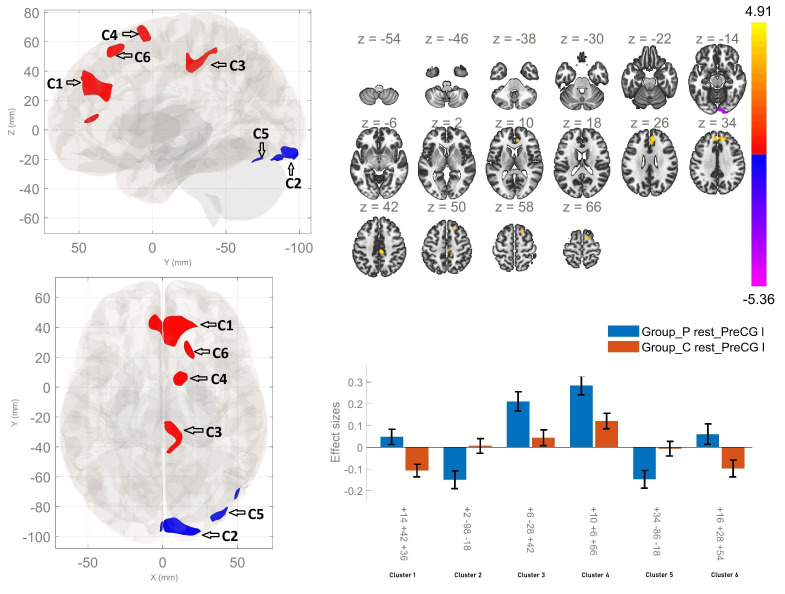
**Resting-state functional connectivity differences of the left precentral gyrus seed between patients with schizophrenia and healthy controls (*p* < 0.05, cluster-level FWE corrected)**. Patients showed increased resting-state functional connectivity (red areas) with the right paracingulate, anterior cingulate, and frontal pole (cluster 1: +14 +42 +36); posterior cingulate, precuneus, right precentral and postcentral gyri (cluster 3: +6 –28 +42); right superior frontal gyrus and supplementary motor area (cluster 4: +10 +6 +66); and right superior frontal gyrus (cluster 6: +16 +28 +54). Decreased resting-state functional connectivity (blue areas) was found with the right occipital pole and lingual gyrus (cluster 2: +2 –98 –18) and right lateral occipital cortex, inferior division, and cerebellar crus I (cluster 5: +34 –86 –18).

Between-group comparisons demonstrated enhanced rsFC between the left PCG and four clusters, the first of which involved the right paracingulate gyrus, the ACC and the right frontal pole. Hyperconnectivity was also observed between the left precentral gyrus and the posterior cingulate region, the precuneus, the right PCG, the right postcentral gyrus, in addition to connectivity between the region of interest and the rSFG, within the patient cohort in comparison of HC. Furthermore, a reduction in the rsFC between the left PCG, the right occipital pole and the right lingual gyrus, along with the right lateral occipital cortex and the right cerebellar crus 1, was established in schizophrenia compared to HC (Fig. 2, Table 3).

**Table 3.** **Functional connectivity differences of the Left Precentral Gyrus seed between patients with schizophrenia and healthy controls**.

Between-group contrast	MNI coordinates x, y, z	Cluster-size	Cluster-threshold (*p < 0.05*, FWE)	Regions within the cluster
P>HC	+14 +42 +36	613	0.0001	Right Paracingulate Gyrus; Anterior Cingulate Gyrus; Right Frontal Pole
	+06 –28 +42	237	0.0001	Posterior Cingulate Gyrus; Precuneus, Right Precentral Gyrus; Right Postcentral Gyrus
	+10 +06 +66	148	0.009	Right Superior Frontal Gyrus; Right Supplementary Motor Area
	+16 +28 +54	115	0.031	Right Superior Frontal Gyrus
HC>P	+02 –98 –18	281	0.0001	Right Occipital Pole; Right Lingual Gyrus
	+34 –86 –18	116	0.029	Right Lateral Occipital Cortex, inferior division; Right Cerebellar Crus 1

## 4. Discussion

The present study detected several significant differences in the rsFC of the right and left precentral gyri in patients with schizophrenia in comparison to HC. First, we observed increased rsFC between the right PCG and three clusters, encompassing bilaterally the SFG, the SMA, and right sided MFG, paracingulate gyrus, and frontal pole, as well as the ACC in the patient group as opposed to the HC. Conversely, we identified hypoconnectivity between the right PCG and the lingual gyrus – bilaterally and the left occipital fusiform gyrus in schizophrenia as compared to HC.

Furthermore, the present study revealed alterations in the rsFC of the other region of interest - the left PCG. First, a significant increase in resting-state functional connectivity was observed between the left PCG and four clusters, including right paracingulate gyrus, ACC, right frontal pole, PCC, precuneus, right pre- and postcentral gyri, rSFG, and rSMA in schizophrenia compared to HC. In addition, the patient group demonstrated hypoconnectivity between the left PCG and the right occipital pole, right lingual gyrus, right lateral occipital cortex, as well as the right cerebellar crus 1. Therefore, we identified a hemispherically specific deficit in rsFC associated with the investigated seed and specific cortical and subcortical regions in schizophrenia.

The current results on the engagement of the frontal lobe in schizophrenia are in accordance with the observations reported in previous research. The frontal lobe contributes to various functions, such as executive, cognitive and attention tasks. A considerable disruption of these functions has been described in individuals suffering from schizophrenia [14]. According to research conducted by Alonso-Solís *et al*. [7] hyperconnectivity has been reported between the dorsomedial prefrontal cortex and the bilateral PCG in patients with AVH compared to NAVH and HC. Moreover, research conducted by Rong *et al*. [15] has demonstrated disturbed inter-network connectivity in schizophrenia. Significantly enhanced inter-network FC was observed between the right and left rostral prefrontal cortex (RPFC) and the bilateral PCG. Additionally, increased connectivity was reported in rMFG and SFG in schizophrenia. The AVH severity was found to be positively correlated with the enhanced connectivity of the rMFG [16]. Taking into account these findings, we suggest that hyperconnectivity between the PCG and the noted regions in the frontal lobe might be implicated in the development of psychotic symptoms, in general and AVH in particular.

The present study also identified increased rsFC between nodes of the sensorimotor network, specifically between the right PCG and the bilateral SMA in schizophrenia. Hyperconnectivity was also observed between the left PCG and the rSMA, as well as the right precentral and postcentral gyri among patients relative to controls. Recent meta-analytic findings from Mo *et al*. [17] reported that the sensorimotor cortex was one of several dysfunctional networks implicated in AVH-state brain alterations. As indicated by the findings of certain study, alterations within the sensorimotor network have been observed during the execution of specific tasks. These observations suggest that the motor cortex undergoes aberrant activations in response to button presses during the presence of AVH [18]. Our findings obtained during resting-state imply the involvement of the sensorimotor network in the mechanism of AVH in the absence of any specific activity.

Another interesting result of our analysis was the enhanced rsFC between both right and left PCG and ACC. As a key node of the SN, impaired functioning of the ACC has been previously implicated in the development of psychosis [19]. It has been suggested that aberrant attribution of salience to external and internal representations may result in the occurrence of delusions and hallucinations. Furthermore, it has been hypothesised that neuronal activity in the ACC may play a mediating role in the manifestation of positive symptoms associated with schizophrenia. Additionally, FC abnormalities involving the ACC have been observed to be correlated with diminished auditory discrimination in individuals diagnosed with schizophrenia [11]. It has been proposed that an imbalance in interaction between SN and SMN may be a contributing factor to the sensory processing anomalies in individuals diagnosed with schizophrenia [15].

According to recent findings reported by Huang *et al*. [20], the detected increase in functional connectivity between the PCG and nodes of the SN in patients with schizophrenia may be interpreted as elevated information processing activity, which may result in emotional and motional processing disruptions in schizophrenia. In contrast to the findings of our study, Amico *et al*. [11] observed hypoconnectivity between the ACC and the PCG in a group of adolescents aged 13 to 16 years who had experienced a definite psychotic episode. A potential factor underlying these divergent outcomes could be that hyperconnectivity between the ACC and the PCG is a characteristic of chronic schizophrenia and AVH and is seen later in the course of the illness as our patients are adults with mean age of 35 years.

Our findings of altered connectivity of the PCG seeds with PCC are in line with the accumulating evidence from neuroimaging studies suggesting altered FC of the DMN in schizophrenia. The results from earlier research have yielded conflicting findings, with some studies reporting an increased FC within the nodes of the DMN, while others have reported hypoconnectivity [21]. Our study identified hyperconnectivity between the PCG and key hubs of the DMN, namely the PCC and the precuneus in the patient group as compared to the HC. Similarly, in a study by Guo *et al*. [22], an increased FC between the PCC/precuneus and the frontal lobe in schizophrenia has been detected. Altered FC of these regions has been demonstrated to result in the impairment of self-referential and introspective processes in schizophrenia [23]. These findings indicate the need for further investigation regarding the functional interactions among the PCC/precuneus, elements of the DMN, and the frontal cortex in the context of schizophrenia pathophysiology.

Structural and functional abnormalities in the cerebellum have been associated with a variety of symptoms in schizophrenia [9, 24]. Although the cerebellum is recognized as being involved in motor functions, including coordination and balance, recent studies utilising FC have revealed its role in more complex associative functions [25]. In a study by Goswami *et al*. [26], hypoconnectivity was detected between parts of the cerebellum and bilateral precentral gyri in schizophrenia. Our analysis showed reduced rsFC between the left PCG and the cerebellar crus 1 in the AVH group. This region is implicated in speech generation and language processing [27]. Therefore, it can be hypothesised that impaired functioning of the cerebellum might contribute to the emergence of psychotic symptoms.

Hypoconnectivity was also observed between the bilateral PCG and the occipital lobe in the AVH group versus the control cohort. Reduced activity in the occipital lobe has been reported during decision-making assignments, episodic memory encoding and recall, as well as during emotion processing tasks in schizophrenia [28]. Research conducted by Yu *et al*. [29] has demonstrated decreased regional homogeneity in bilateral PCG and left middle occipital gyrus during rest in schizophrenia. Moreover, reduced rsFC density was reported between cortical regions involved in processing of sensorimotor and visual information in patients with schizophrenia [30]. However, there is limited research concerning the functional relationship between the PCG and the occipital lobe in psychotic disorders. These findings highlight the need for future research examining precentral-occipital connectivity in relation to schizophrenia and auditory verbal hallucinations.

The schizophrenia group showed hyperconnectivity between the bilateral precentral gyrus and mainly prefrontal regions as opposed to the HC. Another interesting observation was that the pattern of dysconnectivity observed in the study primarily encompassed regions of the right hemisphere, suggesting a predominant involvement of this hemisphere in AVH. As posited by Oertel *et al*. [31], the study demonstrated a negative correlation between reduced laterality and the severity of positive symptoms (assessed by the PANSS). Furthermore, reduced lateralization was found to be negatively associated with the onset of AVH [31]. Prior research has suggested that a decrease in language lateralization may play a role in the development of auditory verbal hallucinations [32]. The present study further supports these findings, demonstrating diminished left>right language asymmetry in individuals experiencing AVH, consistent with patterns reported in the literature.

### Limitations

Several limitations of the present study should be acknowledged when interpreting the findings. First, all patients with schizophrenia were undergoing antipsychotic treatment. While the use of both typical and atypical medications reduces the likelihood that the results are driven by a single drug class, the potential influence of medication on the observed effects cannot be entirely ruled out. Second, the sample size was modest, though comparable to or larger than those in many similar investigations. Finally, only patients experiencing AVH were included, which necessitates careful interpretation of the results within the context of existing research. The additional recruitment of schizophrenia patients with lower level of AVH experienced is to be performed in the near future.

## 5. Conclusions

The present study elucidated notable alterations in resting-state functional connectivity of the right and left PCG in individuals with AVH. Most importantly, the findings of hyperconnectivity of both seeds with nodes of the SN (ACC) and the DMN (precuneus, PCC) contribute to the mounting evidence for the involvement of DMN and SN dysconnectivity in schizophrenia. Moreover, the observed alterations in the rsFC between the bilateral PCG and the frontal and occipital lobe in the patient group might be related to the etiopathogenesis of schizophrenia with a focus on higher hallucination proneness.

## Availability of Data and Materials

The raw data supporting the conclusions of the manuscript will be made available by the authors, without undue reservation, to any qualified researcher.
